# RARRES1 identified by comprehensive bioinformatic analysis and experimental validation as a promising biomarker in Skin Cutaneous Melanoma

**DOI:** 10.1038/s41598-024-65032-1

**Published:** 2024-06-19

**Authors:** Meng Liu, Ruimin Bai, Guanfei Zhang, Xinyi Liu, Ziyang Wang, Ke He, Xinyi Gan, Xiaolin Zhou, Pan Yin, Yan Zheng, Guorong Wang

**Affiliations:** 1 Department of Dermatology, the First Affiliated Hospital of Xi’an Jiaotong University, Xi’an, 710004 Shaanxi China; 2 Center for Mitochondrial Biology and Medicine, The Key Laboratory of Biomedical Information Engineering of Ministry of Education, School of Life Science and Technology, Xi’an Jiaotong University, Xi’an, Shaanxi China; 3 Department of Medicine, Xi’an Jiaotong University, Xi’an, Shaanxi China; 4 Department of General Surgery, ShaanXi Provincial People’s Hospital, Xi’an, 710004 China

**Keywords:** Skin cutaneous melanoma, RARRES1, Tumor suppressor, Autophagy, Melanoma, Cell biology, Cancer, Skin cancer, Tumour biomarkers

## Abstract

Skin cutaneous melanoma (SKCM) is a highly malignant form of skin cancer, known for its unfavorable prognosis and elevated mortality rate. RARRES1, a gene responsive to retinoic acid receptors, displays varied functions in various cancer types. However, the specific role and underlying mechanisms of RARRES1 in SKCM are still unclear. GSE15605 was utilized to analyze the expression of RARRES1 in SKCM. Subsequently, the TCGA and GEO databases were employed to investigate the relationships between RARRES1 and clinicopathological parameters, as well as the prognostic implications and diagnostic efficacy of RARRES1 in SKCM. GO, KEGG, and GSEA analyses were conducted to explore the potential functions of RARRES1. Furthermore, the associations between RARRES1 and immune infiltration were examined. Genomic alterations and promoter methylation levels of RARRES1 in SKCM were assessed using cBioPortal, UALCAN, and the GEO database. Finally, RARRES1 expression in SKCM was validated through immunohistochemistry, and its functional role in SKCM progression was elucidated via in vivo and in vitro experiments. We found that RARRES1 was downregulated in SKCM compared with normal tissues, and this low expression was associated with worse clinicopathological features and poor prognosis of SKCM. The diagnostic efficacy of RARRES1, as determined by ROC analysis, was 0.732. Through GO, KEGG, and GSEA enrichment analysis, we identified 30 correlated genes and pathways that were mainly enriched in the tumor immune microenvironment, proliferation, apoptosis, and autophagy. Additionally, RARRES1 expression was found to be positively related to the infiltration of various immune cells in SKCM, particularly macrophages and T helper cells, among others. Analysis of genomic alterations and promoter methylation revealed that shallow deletion and hypermethylation of the RARRES1 promoter could lead to reduced RARRES1 expression. IHC validation confirmed the downregulation of RARRES1 in SKCM. Moreover, overexpression of RARRES1 inhibited the proliferation and migration of A375 cells, promoted apoptosis, and inhibited autophagic flux. In the mouse xenograft model, RARRES1 overexpression also suppressed SKCM tumor growth. Collectively, these findings suggest that RARRES1 may function as a suppressor and could potentially serve as a prognostic biomarker and therapeutic target for SKCM.

## Introduction

Skin cutaneous melanoma (SKCM) originates from the malignant conversion of melanocytes and is considered the most fatal type of skin cancer. SKCM accounts for approximately 55,500 deaths each year, making up 0.7% of all cancer-related mortalities^1,2^. In recent decades, with the development of immunotherapy and targeted therapies, the mortality rate of SKCM has decreased^3^. However, the prognosis for patients, especially those with metastases, remains formidable, with an approximate 5-year survival rate of only 27%^2^. Moreover, the incidence of malignant melanoma continues to rise in the world wide, with the overall incidence of melanoma in the United States experiencing an annual increase of approximately 1.4% over the past decade^3,4^. Therefore, it is crucial to identify improved prognostic biomarkers and therapeutic targets for SKCM.

Retinoic acid receptor responder 1 (RARRES1), also known as tazarotene-induced gene TIG1, is a retinoid responsive gene that was initially discovered in psoriatic skin raft cultures following retinoid treatment^5^. Research has shown that RARRES1 demonstrates anti-tumor effects in various cancer types. Additionally, hypermethylation of the RARRES1 promoter leads to reduced expression in liver cancer, prostate cancer, breast cancer, and colorectal cancer^6–9^. Notably, RARRES1 plays diverse roles in different tissues. Shyu et al. demonstrated that RARRES1 inhibited cell invasion, migration, and epithelial-mesenchymal transition in testicular cancer cell^10^. They also elucidated the role of RARRES1 in inducing autophagy activity in cervical cancer cells^7^. In addition, aberrant RARRES1 expression can induce activation of M1 microphage in kidney renal clear cell carcinoma and inhibit cells proliferation in colorectal cancer respectively^9,11^. Nevertheless, the expression and functional role of RARRES1 in SKCM remain unclear and need further investigation.

In this study, we comprehensively evaluated RARRES1 expression and its prognostic value in SKCM using data from GEO and TCGA database. Besides, mutation, promoter methylation and potential functions of RARRES1 in SKCM was investigated through bioinformatic analyses. Finally, we confirmed RARRES1 expression in SKCM tissues and experimentally explored its biological roles in cell proliferation, migration, apoptosis and autophagy.

## Material and methods

### Expression of RARRES1 in SKCM and correlation analysis of clinical characteristics

The GSE15605 dataset was downloaded from the Gene Expression Omnibus (GEO, https://www.ncbi.nlm.nih.gov/geo/) database. Using the GEO2R tool, we filtered the expression data of RARRES1 in SKCM and normal samples. The RNAseq data for SKCM was acquired from the TCGA database (https://portal.gdc.cancer.gov) ^12^. We extracted data in TPM format along with clinical information, excluding samples that were normal or lacked clinical information. Subsequently, comprehensive data for 470 SKCM patients was obtained. The entire data processing was carried out using R (version 4.2.1). For data analysis, we employed the stats package and car package, customizing our approach to suit the unique characteristics of the data format. Finally, the data was visualized using the ggplot2 package.

### Prognostic analysis

Patient information and prognosis data of SKCM was obtained from TCGA^12^. Kaplan–Meier survival curves were constructed to compare the prognosis between the high and low RARRES1 groups. Additionally, univariate and multivariate Cox regression analyses were employed to assess the risk factors associated with SKCM. The survival package and survminer package in R (4.2.1) was used for data processing, the ggplot2 package was used for data visualization. The nomogram was constructed and visualized using the R package rms. For the ROC analysis, the expression data of RARRES1 in the GSE15605 dataset was processed by Graphpad prism.

### Correlated genes enrichment analysis

The mRNA expression data of 470 SKCM patients in TCGA database was obtained. Pearson correlation was used to identify genes associated with RARRES1 expression. The results were filtered, selecting the top 15 genes with the highest positive correlation and the top 15 genes with the highest negative correlation. R package ggplot2 was used to create a co-expression heatmap. Then, we selected 900 genes which were significantly correlated with RARRES1 (*P* < 0.05, |R|> 0.5) to perform GO and KEGG analysis (Table S1). GO analysis is a widely accepted approach for performing comprehensive functional enrichment studies on a large scale, encompassing biological processes (BP), molecular functions (MF), and cellular components (CC)^13^. KEGG serves as a highly utilized database for archiving data related to genomes, biological pathways, diseases, and pharmaceuticals^14^. GO annotation analysis and KEGG pathway enrichment analysis of correlated genes were performed using R package clusterProfiler. *P* < 0.05 was considered statistically significant. Results of enrichment analysis were visualized using ggplot2, igraph, and ggraph.

### GSEA analysis

Gene Set Enrichment Analysis (GSEA) is a powerful analytical technique designed to assess whether a predefined group of genes exhibits statistically significant and coherent variations between two distinct phenotypes^15^. The RNAseq data of 470 SKCM patients was analyzed by DESeq2 and edgeR. After obtaining gene names and their corresponding log fold changes (logFC), we performed GSEA using the clusterProfiler. The objective was to unveil substantial disparities in functional and pathway profiles between the high- and low-RARRES1 groups. h.all.v2022.1.Hs.entrez.gmt [Hallmarks] in MSigDB Collections (https://www.gsea-msigdb.org/gsea/msigdb/collections.jsp) was used as the reference gene collection. An adjusted *P* < 0.05 and False discovery rate (FDR) < 0.25 were considered as significant enrichment.

### Correlation analysis between RARRES1 expression and immune infiltration

For the information of 470 SKCM patients in TCGA database, immune infiltration analysis was performed using the ssGSEA algorithm based on that provided in the R package GSVA [1.46.0], using markers of 24 immune cells to calculate the immune infiltration corresponding to the cloud-based data^16,17^. The data were processed using R (4.2.1) and correlation analysis was performed between the primary variables and immune infiltration matrix data, and the results were displayed as lollipop plots and analyzed through Spearman correlation analysis. Data was visualized using ggplot2.

### Analysis of gene mutations and promoter methylation analysis

cBioPortal database (http://cbioportal.org) was used to analyze the profile of genetic alterations in SKCM, amino acid changes in proteins, and the effect of different copy number variants (CNV) on RARRES1 mRNA expression^18^. Furthermore, UALCAN database (https://ualcan.path.uab.edu/) was utilized to assess RARRES1 promoter methylation in SKCM^19^. Finally, we downloaded GSE120878 dataset from the GEO database. This dataset consists of DNA methylation data for 89 SKCM and 73 nevi. Furthermore, EWAS database (https://ngdc.cncb.ac.cn/ewas/) was used to retrieve methylation sites specifically associated with RARRES1 in SKCM.

### Clinical samples

Paraffin-embedded skin samples were taken from 20 patients (11 male and 9 female, aged 57–74 years) diagnosed with SKCM and 11 healthy controls (6 male and 5 female, aged 60–78 years) diagnosed with intradermal nevus. These samples were sourced from the tissue bank of the First Affiliated Hospital of Xi’an Jiaotong University. All diagnoses were confirmed by two experienced pathologists. Written informed consent was diligently obtained from all patients before sample collection and analysis. Ethics approval for the study was acquired from the Institutional Ethics Committee of Xi’an Jiaotong University.

### Immunohistochemistry

Immunohistochemistry was performed according to a standard procedure. The RARRES1 antibody (ab198908, Abcam) and Ki-67 antibody (GB111141, Servicebo, China) was used. RARRES1 sections were semi-quantitatively categorized into four grades: negative (-), weak ( +), moderate (+ +), and strong (+ + +). The results were independently evaluated by two pathologists. Ki67 sections were scanned by Hamamatsu digital pathology. Image J software was used to quantify the Ki67 staining.

### Cell culture

The A375 cells were purchased from ATCC and cultured in Dulbecco’s modified Eagle’s medium containing with 10% fetal bovine serum, penicillin (100 U/mL), and streptomycin (100 μg/mL) at 37℃ in a humidified atmosphere with 5% CO_2_.

### Plasmid construction and transient transfection

The RARRES1 expression plasmid was synthesized by MiaoLing Plasmid (Wuhan, China). A375 cells were transfected with 2 μg of the RARRES1 expression plasmid (pCMV-RARRES1(human)-3×FLAG) or the control pCDNA vector using PEI for 48 h. The transfection procedures were conducted according to the standard protocols.

### Lentivirus production and construction of stable RARRES1-overexpressing A375 cell line

Full length cDNA encoding human RARRES1 was inserted into pLV3-CMV-RARRES1(human)-3 × FLAG-CopGFP-Puro to obtain RARRES1-overexpressing plasmid pCDH-RARRES1. pCDH-RARRES1 lentivirus particles were obtained by co-transfecting pCDH-RARRES1 with packaging plasmids (pLP1, pLP2 and pLP-VSVG) into HEK293T cells. A375 cells were infected with concentrated pCDH-RARRES1 lentivirus particles and then were screened by puromycin to obtain stable RARRES1-overexpressed A375 cell line.

### Cell proliferation assay

The proliferation rates of A375 cells transfected with pcDNA3.4-Vector or pCDNA3.4-RARRES1 were assessed by MTT reagent (3-(4,5-dimethylthiazol-2-yl)-2,5-diphenyltetrazolium bromide). These assays were performed according to the manufacturer’s instructions.

### Crystal violet staining

A total of 500 cells, stably expressing RARRES1 or Vector, were seeded into 6 wells plates and cultured for 2 weeks. Colonies were fixed and stained by crystal violet according to the standard protocols.

### Soft agar clonogenic assay

The cells were trypsinised, collected after puromycin screening, and counted using a haemocytometer. Furthermore, 200 cells/well were seeded in 0.7% soft agar in media on a 12-well plate, over a 1.2% soft agar underlay. Finally, 1 mL normal medium with was added to cover the top agar. They were grown for about 10–14 days and cell clone images were captured by a Nexcope NIB620 inverted microscope (Nexcope, Ningbo, China).

### Cell cycle assay

After transfected by pcDNA3.4-RARRES1/Vector for 48 h, cells were collected and washed with PBS, then cells were fixed with 70% ethanol at − 20 °C overnight. 100 μL RNase A solution was added and cells were incubated at 37 °C for 30 min. Finally, 400 μL propidium iodide (PI) solution was added and incubated at room temperature for 30 min in the dark. The DNA content was detected using flow cytometry (NovoCyte flow cytometry, ACEA Biosciences).

### Transwell assay

Transwell assays were conducted using Transwell chambers (3422; Corning). The lower chamber was filled with 800 μL of complete culture. In the upper chamber, 3 × 10^4^ cells were seeded using 200 μL of fetal bovine serum-free medium. After a 24-h incubation, the migrating cells were fixed and then stained with 0.1% aqueous crystal violet. Microscopic images were captured, and the cell count in each field was documented.

### Apoptosis assays

Cells transfected by pcDNA3.4-RARRES1/Vector, were stained with FITC Annexin V Apoptosis Detection Kit according to the manufacturer’s instructions. Cells apoptosis was analyzed by flow cytometry (NovoCyte flow cytometry, ACEA Biosciences).

### Protein extraction and Western blotting

Western blotting was performed as described previously^20^. The following antibodies were used in this study: RARRES1 (MA5-26,247, Invitrogen), Flag (66,008-4-Ig, proteintech), P53(CST# #9282), BAX (50,599-2-Ig, proteintech), BAK (29,552-1-AP, proteintech), BCL-2 (12,789-1-AP, proteintech), Cyclin D1 (ab16663), Cyclin E1 (11,554-1-AP, proteintech, China), P62 (CST#23,214), LC3I/II (CST#4108), β-actin (CST#3700), GAPDH (CST#5174), β-Tubulin (M30109, Abmart, China). The membranes were incubated with HRP conjugated secondary antibody at room temperature for 1 h. Finally, the chemiluminescent signal was detected using the ultrasensitive ECL kit (Pierce Chemical, Rockford, IL, USA). The intensity of the bands was quantified by using Image J software (National Institutes of Health, Bethesda, MD, USA).

### ROS determination

ROS levels were measured with DHE (Biyotime, #S0063) dyes. A375 cells were first seeded into 12-well plates overnight and transfected with pcDNA3.4-Vector or pCDNA3.4-RARRES1 for 48 h. Then, cells were incubated with DHE solution dissolved in FBS-free medium for 45 min and then washed with PBS. Nexcope NIB620 inverted microscope (Nexcope, Ningbo, China) was used to take the fluorescent images. Image J software was usd to analyze DHE relative fluorescence intensity. (bar length = 50 um).

### LysoSensor green DND-189 staining

Lysosomal staining with LysoSensor Green DND-189, was performed according to the manufacturer (Yeasen 40767ES50, Shanghai, China). After transfection of pCDNA3.4, Lysosensor green was added to the medium and incubated for 10 min at 37℃, with 5% CO_2._ Then cells were directly transferred to a fluorescent microscope for imaging.

### Xenograft tumor model

A total of 4 × 10^6^ cells transfected with pCDH-RARRES1/Vector were subcutaneously injected into4-week-old female BALB/c-nude mice on their right flanks (6 mice/group). Tumor volume was calculated every 3 days. On day 10, the mice were sacrificed and subcutaneous tumors were collected. Tumor volumes were calculated using the formula V = 1/2 × L × W^2^, where "L" represents tumor length and "W" represents tumor width. All animal experiment were approved by the Institutional Animal Care and Use Committee of Xi’an Jiaotong University.

### Statistical analysis

T-test was used to analyze the differential expression genes between SKCM and normal. Kruskal–Wallis Test, Welch t' test and Wilcoxon rank sum test were used to assess the differences in RARRES1 expression among samples with varying clinical features. Logrank test was used to compare the prognosis between the high and low expression groups of RARRES1. Sperman’s correlation was carried out to evaluate enrichment scores of 20 immune cells and RARRES1 expression separately. Experimental data were expressed as the mean ± SEM. Statistical graphs and analyses were conducted using GraphPad Prism software. To compare two unpaired groups, Student's t-test was employed, and *P* < 0.05 was deemed as statistically significant.

### Ethics approval and consent to participate

Ethics approval for the study was acquired from the Institutional Ethics Committee of Xi’an Jiaotong University, and the use of animals in our experiments was consistent with ethical requirements.

## Results

### Expression, clinicopathological correlation, prognostic and diagnostic potential of RARRES1 in SKCM

**Figure 1:**
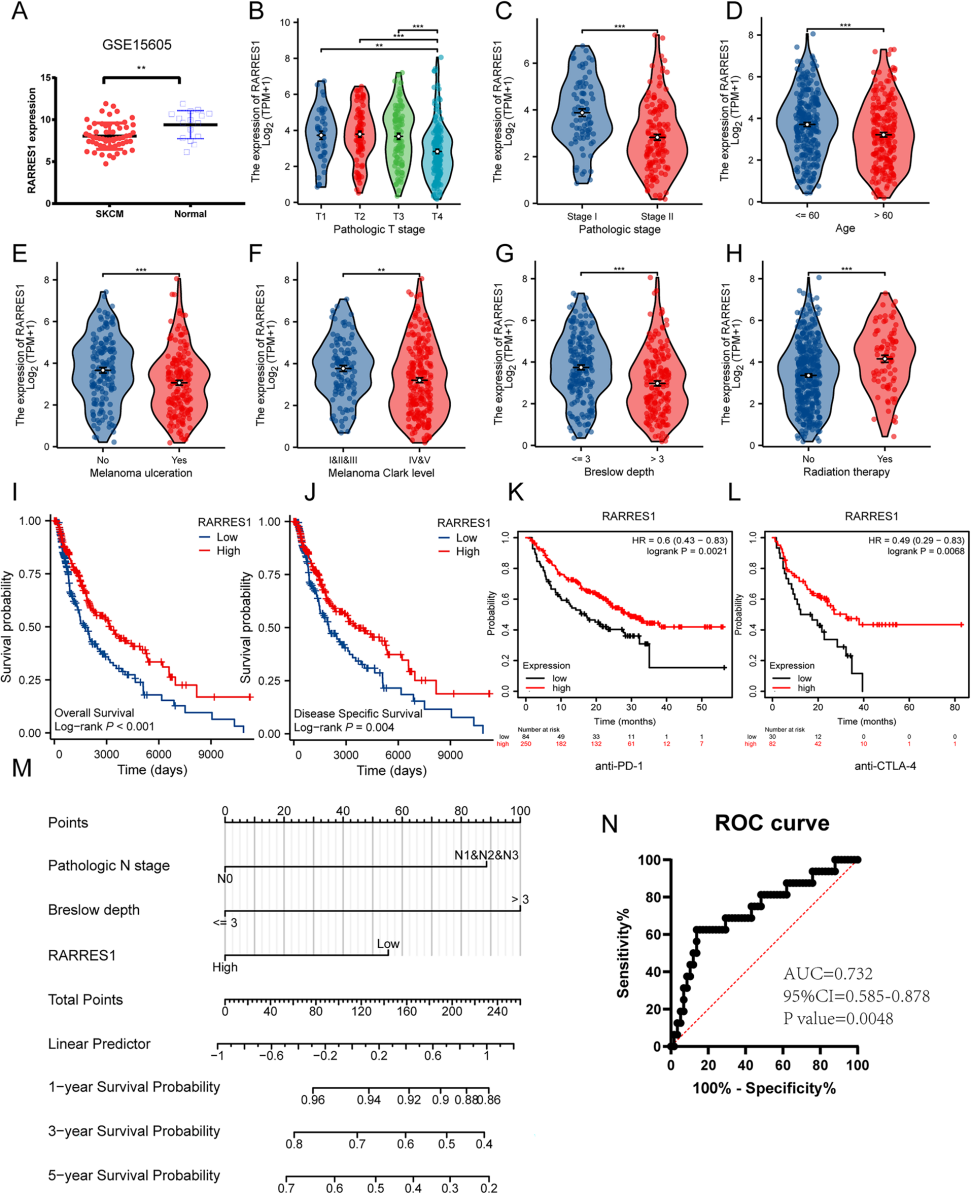
Expression, clinicopathological correlation, prognostic and diagnostic potential of RARRES1 in SKCM. (**A**) RARRES1 expression in 58 SKCM tissues and 16 normal tissues in the GSE15605 dataset. (**B**–**H**) The association of RARRES1 expression and (**B**) T classification, (**C**) pathological stages, (**D**) patient’s age, (**E**) melanoma ulceration, (**F**) melanoma Clark level, (**G**) Breslow depth, (**H**) radiation therapy. (**I**–**J**) Survival curves of OS and DSS. (**K**–**L**) The prognostic impact of RARRES1 on immunotherapy with anti-PD-1 treatment and anti-CLTA-4 treatment. (**M**) Nomogram that integrates RARRES1 expression and other prognostic factors in SKCM. (**N**) ROC curve for evaluating the diagnostic value of RARRES1. **P < 0.01 and ***P < 0.001.

The expression of RARRES1 in SKCM (n = 58) and normal tissues (n = 16) was analyzed using GSE15065 dataset. As shown in Fig. 1A, RARRES1 expression in SKCM was significantly down-regulated compared to the normal tissues. Subsequently, TCGA database was used to investigate the correlation between RARRES1 expression and clinical characteristics of SKCM. We found that RARRES1 was significantly reduced in SKCM patients of T4 stage, pathological stage II, age over 60, with melanoma ulcer, higher Clark level, higher Breslow thickness and without radiotherapy (Fig. 1B-H), indicating decreased RARRES1 expression was associated with poor clinical features of SKCM. We then assessed the correlation between RARRES1 expression and survival of SKCM patients. The Kaplan–Meier survival analysis indicated that low expression of RARRES1 was associated with unfavorable overall survival (OS) or disease specific survival (DSS) (Fig. 1I–J). Besides, RARRES1 expression significantly affected the OS of SKCM patients undergoing immunotherapy, showing an overall correlation with poor OS after immunotherapy (HR = 0.6 (0.43–0.83), logrank *P* = 0. 0021 for all anti-PD-1 treatment; HR = 0.049 (0.29–0.83), logrank *P* = 0.0068 for anti-CTLA-4 treatment) (Fig. 1K-L). We futher evaluated the factors correlated with OS in SKCM, as shown in Table 1. Univariate Cox regression analysis showed that tumor stage (T, N, M), patient age, ulceration, Clark level, Breslow depth, and RARRES1 expression were associated with survival. Multivariate Cox regression analysis demonstrated that only pathological N stage, Breslow depth and RARRES1 are independent prognostic factors for survival. For further verification, we constructed a nomogram of OS that incorporated RARRES1, pathological N stage and Breslow depth, enabling us to predict the 1-, 3-, 5-year survival probability in SKCM (Fig. 1M). Based on the expression data of RARRES1 in GSE15605, the diagnostic value of RARRES1 in SKCM was calculated, the area under the curve (AUC) was 0.732 (*P* = 0.0048), which illustrated that RARRES1 had a good diagnostic performance in SKCM^21^ (Fig. 1N).

**Table 1:** The univariate and multivariate analyses of overall survival.

Characteristics	Number (n)	Univariate analysis		Multivariate analysis	
		HR (95% CI)	*P*-value	HR (95% CI)	*P*-value
T stage (T1-2 vs. T3-4)	362 (119 vs. 243)	2.099 (1.511–2.914)	< 0.001	0.857 (0.484–1.517)	0.596
N stage (N0 vs. N1-3)	403 (225 vs. 178)	1.760 (1.310–2.365)	< 0.001	2.432 (1.623–3.646)	< 0.001
M stage (M0 vs. M1)	431 (407 vs. 24)	1.902 (1.032–3.506)	0.039	2.059 (0.822–5.157)	0.123
Age (< = 60 vs. > 60)	457 (247 vs. 210)	1.663 (1.256–2.201)	< 0.001	1.197 (0.808–1.772)	0.370
Melanoma ulceration (No vs. Yes)	314 (147 vs. 167)	2.099 (1.506–2.927)	< 0.001	1.431 (0.949–2.160)	0.088
Clark level (I&II&III vs. IV&V)	316 (96 vs. 220)	2.184 (1.520–3.137)	< 0.001	1.307 (0.801–2.133)	0.284
Breslow depth (< = 3 vs. > 3)	356 (181 vs. 175)	2.665 (1.948–3.644)	< 0.001	1.922 (1.142–3.238)	0.014
RARRES1 (High vs. Low)	457 (229 vs. 228)	1.633 (1.247–2.139)	< 0.001	1.603 (1.095–2.347)	0.015

### Gene correlation and functional enrichment analysis of RARRES1 expression in SKCM

**Figure 2:**
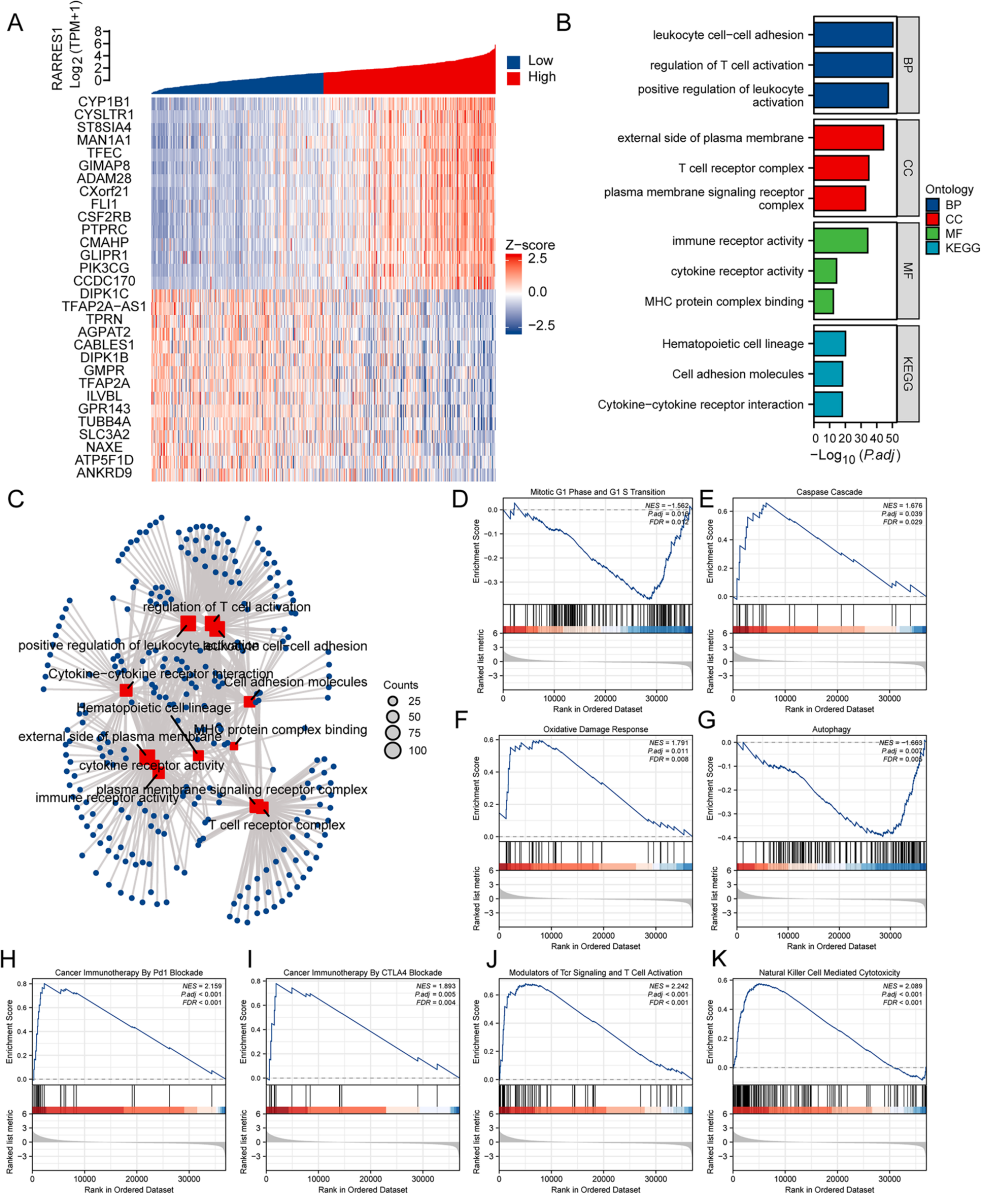
Gene correlation and functional enrichment analysis of RARRES1 expression in SKCM. (**A**) Top 30 genes correlated with RARRES1 expression. (**B**) The GO and KEGG enrichment analysis of RARRES1. (**C**) The network results of GO and KEGG pathways. (D-K) GSEA analysis on (**D**) mitotic G1 phase and G1/S transition, (**E**) caspase cascade, (**F**) oxidative damage response, (**G**) autophagy, (**H**) cancer immunotherapy by PD1 blockade, (**I**) cancer immunotherapy by CTLA4 blockade, (**J**) modulators of TCR signaling and T cell activation, (**K**) natural killer cell mediated cytotoxicity.

We proceeded to employ bioinformatics analysis in order to examine the biological function of RARRES1. Initially, we conducted correlation analysis between RARRES1 and other genes in the TCGA database. Subsequently, we assigned ranks to genes that exhibited a significant correlation with RARRES1 expression (*P* < 0.05). Consequently, we identified the top 15 genes that displayed a positive correlation and the top 15 genes that displayed a negative correlation with RARRES1, as visually depicted in the heatmap (Fig. 2A). The GO terms were classified into BP, CC and MF. Within the BP category, the enriched terms included leukocyte cell–cell adhesion, regulation of T cell activation, and positive regulation of leukocyte. In the CC category, the enriched terms included external side of the plasma membrane, T cell receptor complex, and plasma membrane signaling receptor complex. Lastly, in the MF category, the enriched terms included immune receptor activity, cytokine receptor activity, and MHC protein complex binding. In terms of KEGG pathway enrichment, significant enrichments were observed in hematopoietic cell lineage, cell adhesion molecules, and cytokines-cytokine receptor interaction (Fig. 2B-C). We also performed a GSEA analysis to identify the key pathways related to RARRES1. GSEA analysis found that 480 data sets satisfied the criteria of an FDR < 0.25 and a *P* < 0.05, as shown in Table S2. The enriched pathways encompassed various processes, including mitotic G1 Phase and G1/S transition (Fig. 2D), caspase cascade (Fig. 2E), oxidative damage response (Fig. 2F), autophagy (Fig. 2G), cancer immunotherapy by PD1 blockade (Fig. 2H), cancer immunotherapy by CTLA4 blockade (Fig. 2I), modulators of Tcr signaling and T cell activation (Fig. 2J), natural cell mediated cytotoxicity (Fig. 2K) and so on.

### Correlation between RARRES1 expression and immune cell infiltration in SKCM

**Figure 3:**
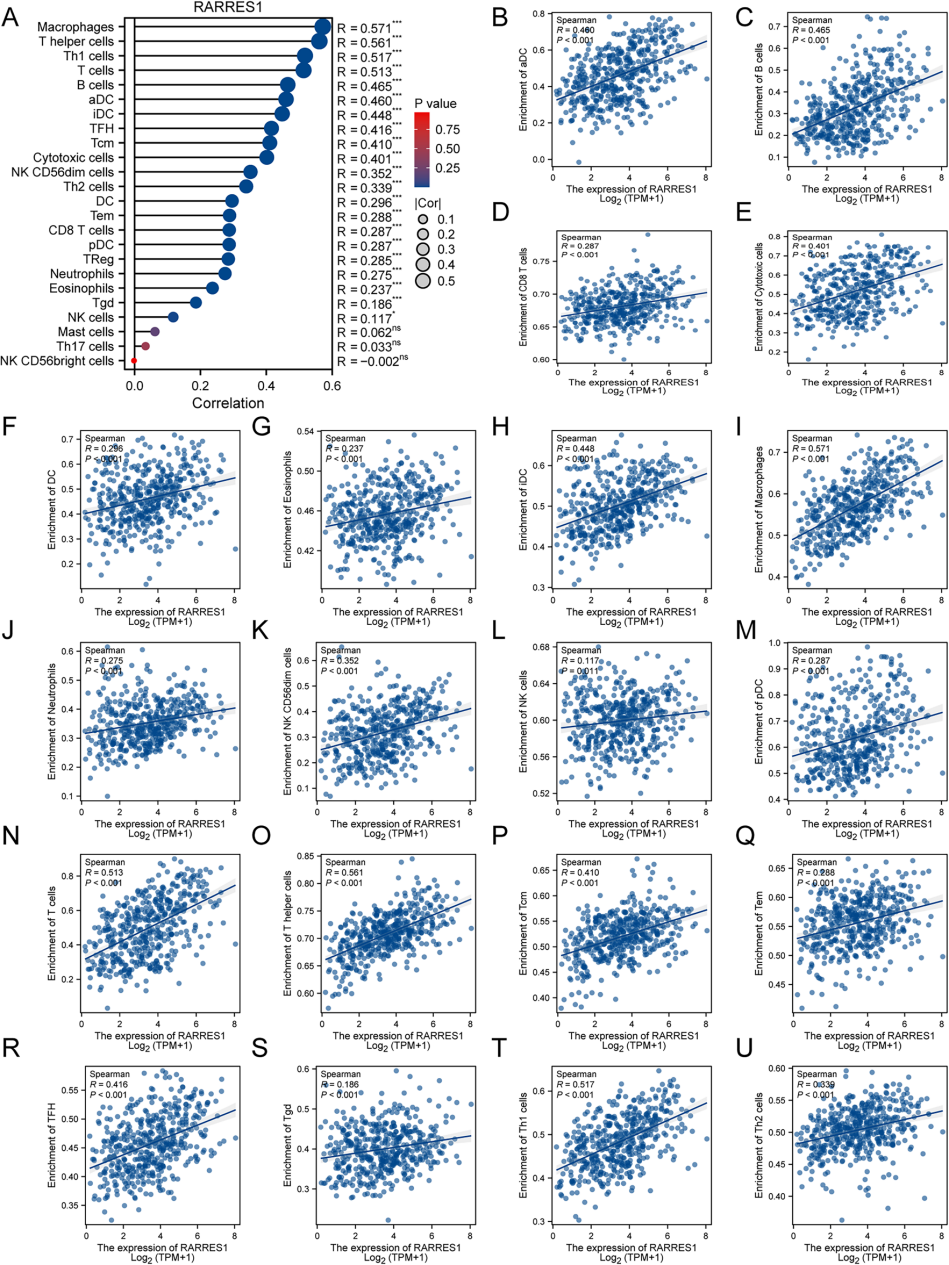
RARRES1-related immune infiltration in SKCM. (**A**) Lollipop chart of the correlation between RARRES1 expression and 24 types of immune cells. (**B**–**U**) Scatter plot of GPR143 expression and immune cell infiltration: (**B**) activated dendritic cells, (**C**) B cells, (**D**) CD8 + T cells, (**E**) cytotoxic cells, (**F**) dendritic cells, (**G**) eosinophils, (**H**) immature dendritic cells, (**I**) Macrophages, (**J**) neutrophils, (**K**) NK CD56dim cells, (**L**) NK cells, (**M**) plasmacytoid dendritic cells, (**N**) T cells, (**O**) T helper cells, (**P**) T central memory cells, (Q) T effector memory cells, (**R**) T follicular helper cells, (**S**) T gamma delta cells, (**T**) Th1 cells, (**U**) Th2 cells. *P < 0.05, ***P < 0.001, ns: no significance.

Functional enrichment analysis of RARRES1 revealed that numerous pathways were closely associated with immune processes. Furthermore, tumor-associated immune cells play important roles in determining tumor progression, and the infiltration of immune cells is strongly related to the outcome of the tumor^22^. Therefore, we assessed the correlation between the RARRES1 expression and the extent of immune cell infiltration in SKCM. As shown in Fig. 3A, RARRES1 expression in SKCM was positively correlated with the infiltration of various immune cells, indicating the low expression of RARRES1 might be unfavorable for immune cell infiltration in SKCM. We further compare the difference in immune cell infiltration between RARRES1 high and low expression groups. The results demonstrated that SKCM with low expression of RARRES1 had less immune cell infiltration (Fig. 3B-U).

### Mutation and promoter methylation of RARRES1 in SKCM

**Figure 4:**
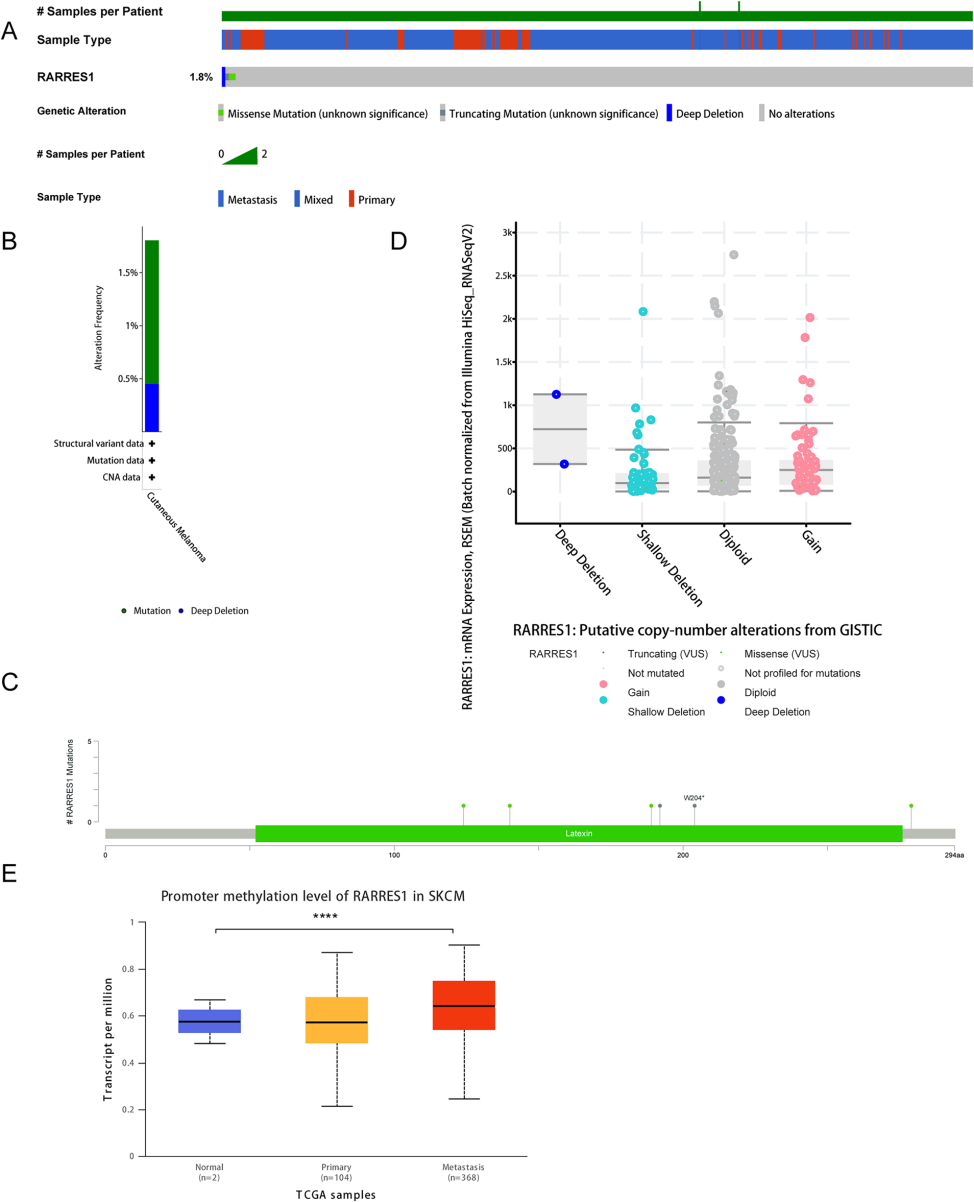
Mutation and promoter methylation of RARRES1 in SKCM. (**A**) Mutation of SKCM in 443 SKCM samples. (**B**) Mutation frequency distribution of RARRES1 in SKCM. (**C**) 6 mutated nucleotide positions in RARRES1 gene. (**D**) RARRES1 expression in different RARRES1 CNV groups. (**E**) Promoter methylation level of RARRES1 in normal tissues, primary SKCM, and metastatic SKCM. **** *P* < 0.0001.

**Figure 5:**
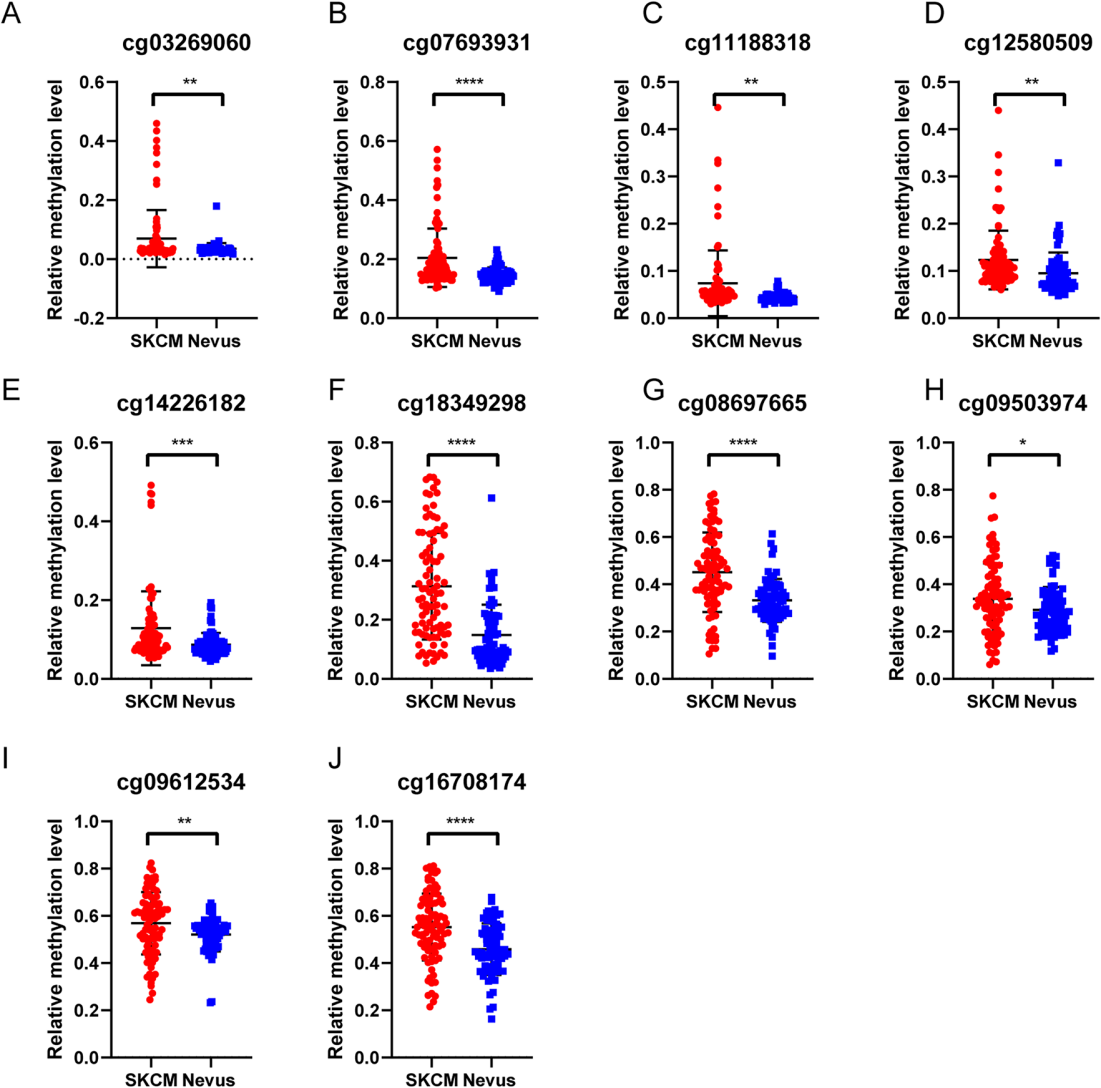
10 high promoter methylation sites in SKCM. (**A**) cg03269060; (**B**) cg07693931; (**C**) cg11188318; (**D**) cg12580509; (**E**) cg14226182; (**F**) cg18349298; (**G**) cg08697665; (**H**) cg09503974; (**I**) cg09612534; (**J**) cg16708174. *P < 0.05, **P < 0.01, ***P < 0.001, **** *P* < 0.0001.

Previous studies have demonstrated that the abnormal expression of RARRES1 arises due to hypermethylation of the CpG island associated with the RARRES1 promoter^23^. Thus, we analyzed the genomic alteration and promoter methylation of RARRES1 via cBioPortal, UALCAN and GEO database. In a total of 443 SKCM samples, the mutation rate of RARRES1 was found to be 1.8% (Fig. 4A). This implies that there were 8 instances of gene alteration, comprising 6 cases of mutation and 2 cases of deep deletion (Fig. 4B). More specifically, the missense mutation of RARRES1 in SKCM led to changes in the amino acids that constitute the protein (Table 2). All 6 mutated nucleotide positions were visually represented in Fig. 4C. Copy number variations (CNV) involve the amplification or deletion of chromosomal segments resulting from genomic rearrangements. CNVs play a crucial role in influencing gene expression^24^. We then investigated the association between the RARRES1 expression and CNVs. As shown in Fig. 4D, shallow deletion maybe responsible for the downregulated of RARRES1. In addition, data from UALCAN database showed that the promoter methylation of metastatic melanoma was significantly higher than that of the normal group, as shown in Fig. 4E. However, due to the limited sample size of the normal group, we were unable to identify any significant differences in the promoter methylation levels between normal and SKCM tissues. In order to conduct a more comprehensive analysis of the promoter methylation of RARRES1 in SKCM, we identified 15 promoter methylation sites of RARRES1 based on the GSE120878 dataset. Among these sites, 10 exhibited significantly higher levels of methylation in SKCM compared to nevus (Fig. 5A-J). These findings suggest a strong correlation between the downregulation of RARRES1 expression in melanoma and the presence of mutations as well as elevated promoter methylation.

**Table 2:** Mutated nucleotide position of RARRES1 in SKCM.

Sample ID	TCGA-D3-A51T-06	TCGA-GN-A266-06	TCGA-EE-A29M-06	TCGA-EB-A41A-01	TCGA-EB-A431-01	TCGA-FR-A8YC-06
Cancer type	SKCM	SKCM	SKCM	SKCM	SKCM	SKCM
Protein Change	G279R	W192*	G124E	T140A	W204*	R189K
Mutation Type	Missense	Nonsense	Missense	Missense	Nonsense	Missense
Variant Type	SNP	SNP	SNP	SNP	SNP	SNP
Copy #	Diploid	Diploid	Gain	Blanks	Blanks	Shallow Deletion
Chromosome	3	3	3	3	3	3
Start position	158,415,517	158,422,676	158,428,691	158,428,644	158,422,641	158,422,686
End position	158,415,517	158,422,676	158,428,691	158,428,644	158,422,641	158,422,686
Allele Freq (T)	0.3	0.31	0.44	0.47	0.11	0.3
# Mut in Sample	978	3271	1959	1531	1398	2480

### Decreased RARRES1 expression was confirmed in SKCM by immunohistochemistry

**Figure 6:**
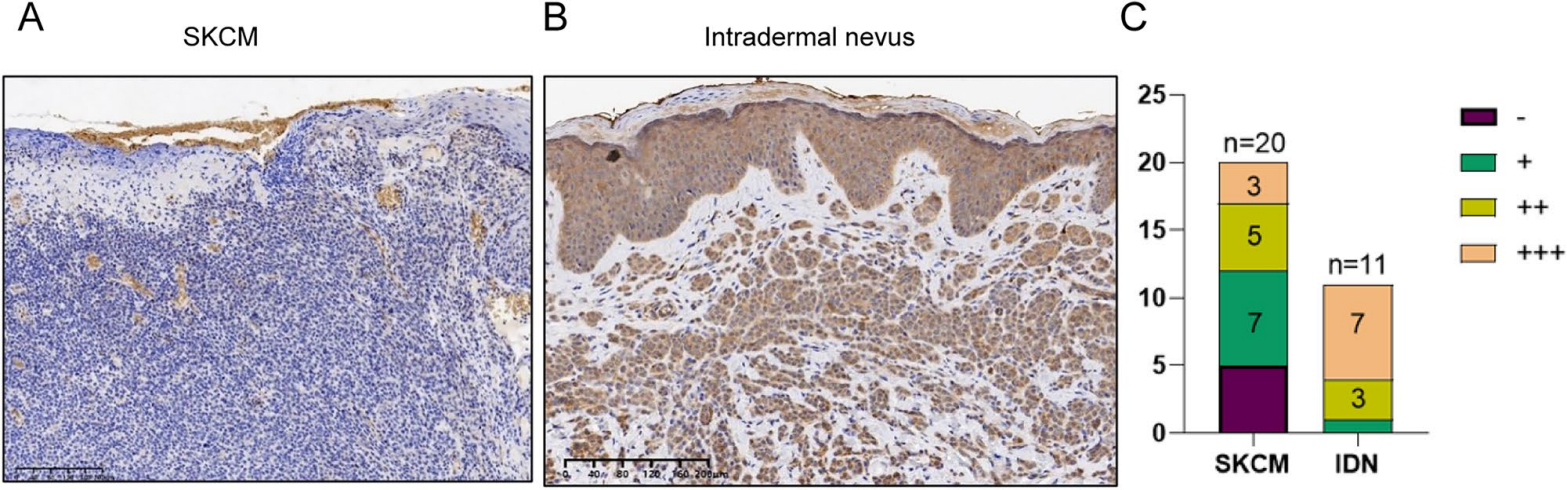
Analysis of RARRES1 expression in SKCM and intradermal nevus (IDN) tissues. (**A**) Immunohistochemical staining of RARRES1 in SKCM (bar length = 200 μm). (**B**) Immunohistochemical staining of RARRES1 in intradermal nevus (bar length = 200 μm). (**C**) Semiquantitative analysis of RARRES1 staining.

To validate the expression of RARRES1, a total of 20 skin samples from patients with SKCM and 11 skin samples from individuals with intradermal nevus were collected for immunohistochemistry staining. As depicted in Fig. 6A, RARRES1 was found to be weakly expressed in the tumor tissues of SKCM. In contrast, a noticeable increase in the expression of RARRES1 was observed in intradermal nevi (Fig. 6B). Furthermore, a semiquantitative analysis demonstrated a significant downregulation of RARRES1 in SKCM (Fig. 6C).

### RARRES1 overexpression inhibits A375 cell proliferation and migration

**Figure 7:**
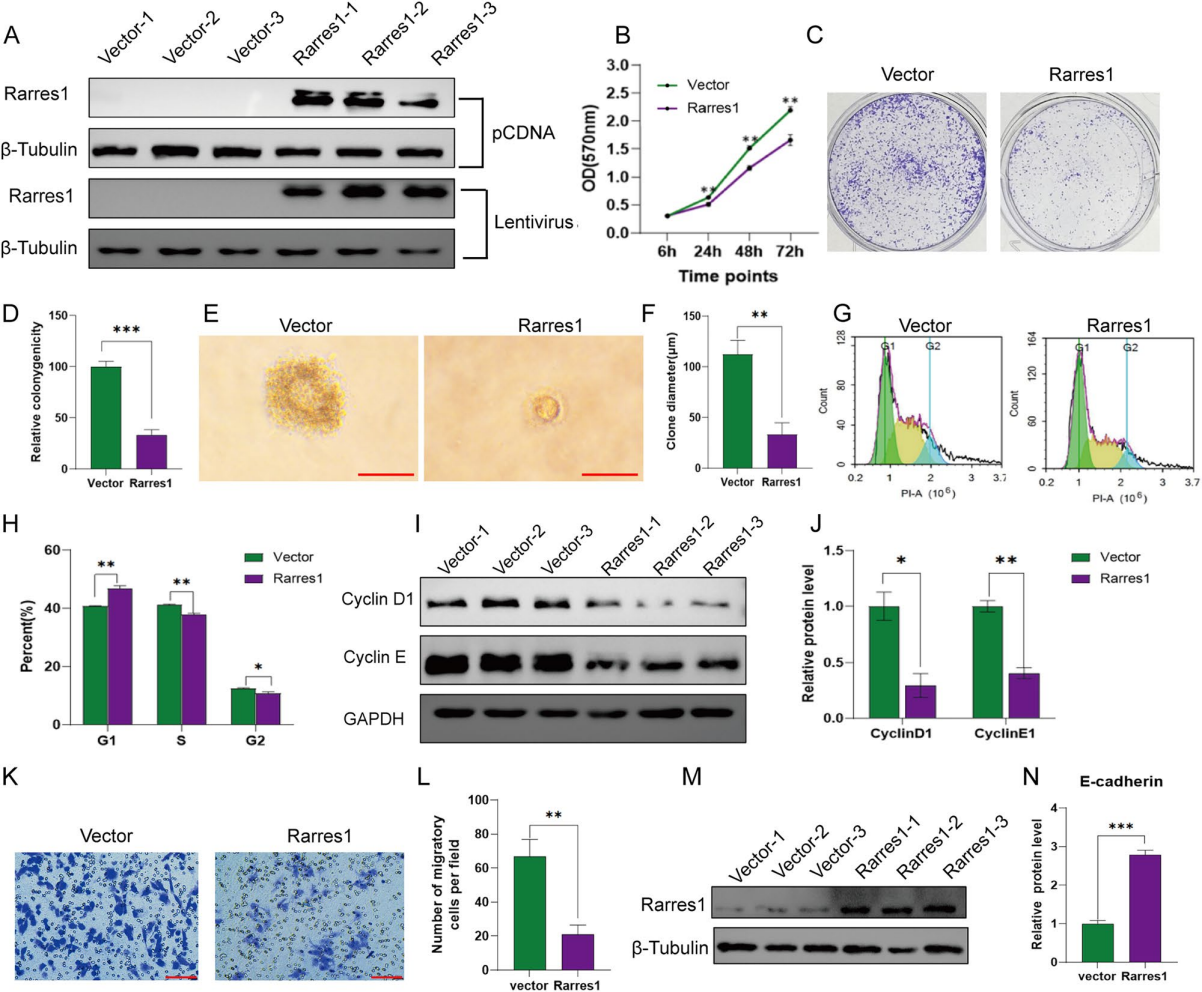
Effects of RARRES1 overexpression on A375 cell proliferation and migration. (**A**) Relative protein levels of RARRES1 in A375 cells transfected with pCDNA-RARRES1 of Lentivirus-RARRES1. (**B**) MTT assay at 6, 24, 48 and 72 h after RARRES1 overexpression. (**C**–**D**) Colony forming assays after RARRES1 overexpression. (**E**–**F**) Soft agar assays after RARRES1 overexpression (bar length = 1 mm). (**G**–**H**) Cell cycle analysis after RARRES1 overexpression. (I-J) Western blotting analysis of cell cycle regulators after over expression. (**K**–**L**) Transwell migration assay after RARRES1 overexpression (bar length = 100 μm). (**M**–**N**) Western blotting analysis of E-cadherin after RARRES1 overexpression. *P < 0.05, **P < 0.01, ***P < 0.001. All assays were performed in triplicate.

Based on the above results, we suppose that Rarres1 is a tumor suppressor in SKCM. Furthermore, we conducted gain-of-function assays in A375 cells by transfected pCDNA-RARRES1/Vector and lentiviruses-RARRES1/Vector. The overexpressing efficiency is shown in Fig. 7A. Results from MTT assay (Fig. 7B) showed that RARRES1 overexpression inhibited A375 cell proliferation. Consistently, crystal violet staining (Fig. 7C-D) and soft agar assays (Fig. 7E-F) showed an injured ability of colony formation on RARRES1 upregulated A375 cells. Flow cytometry analysis was subsequently conducted to assess the impact of RARRES1 on the cell cycle distribution of A375 cells. As demonstrated in Fig. 7G-H, RARRES1 overexpression resulted in an increased cell population in the G0/G1 phase and a concurrent decrease in the S phase. In addition, we detected the expression of cell cycle related protein. Western blotting results revealed that RARRES1 overexpression led to a reduction in the expression levels of Cyclin D1 and Cyclin E1, as depicted in Fig 7 I and J. As the pathway related to migration and invasion is visible in the enrichment analysis, we next analyzed the role of RARES1 on the migration or invasion of A375 cells. We directly studied in vitro migration in 24-well plates inserted with 8.0-mm-pore transwells. Significant decrease in migration was observed in RARRES1 overexpression cells compared with vector group (Fig. 7K-L). Consistently, an increased expression of E-cadherin was observed upon RARRES1 overexpression (Fig. 7M-N). Overall, these findings indicate that RARRES1 plays an important role in cell proliferation, colony formation, cell cycle and migration, and its overexpression significantly suppresses A375 cell proliferation and migration.

### Overexpression of RARRES1 promotes apoptosis and inhibits autophagic flux in A375 cell line

**Figure 8:**
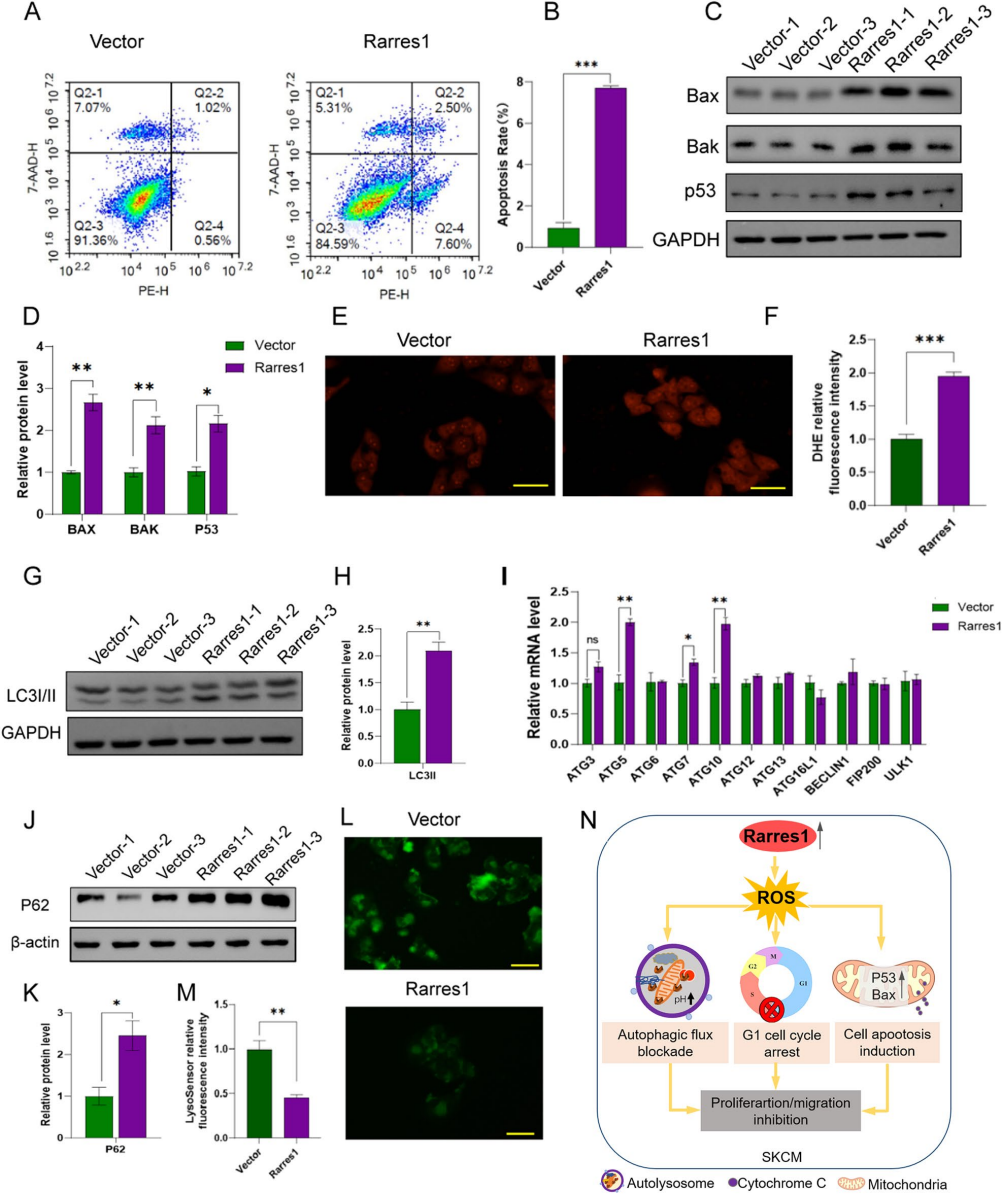
Effects of RARRES1 overexpression on A375 cell apoptosis and autophagy. (**A**–**B**) Cell apoptosis assay after RARRES1 overexpression. (**C**–**D**) Western blotting analysis of cell apoptosis biomarkers after RARRES1 overexpression. (**E**) Representative images and (**F**) quantification of ROS in A375 cells measured by DHE dye after RARRES1 overexpression (bar length = 50 μm). (**G**–**H**) Western blotting analysis of LC3I/II after RARRES1 overexpression. (**I**) Relative mRNA levels of autophagy related genes after RARRES1 overexpression. (**J**–**K**) Western blotting analysis of P62 after RARRES1 overexpression. (**L**) Representative images and (**M**) quantification of lysosomal pH in A375 cells determined by lysosensor Green DND-189 after RARRES1 overexpression (bar length = 50 um). (**N**) Schematic diagram illustrating the mechanism by which RARRES1 inhibits proliferation and migration of SKCM cells. *P < 0.05, **P < 0.01, ***P < 0.001. All assays were performed in triplicate.

We then investigated the role of RARRES1 in the regulation of cell apoptosis. A375 cells transfected with the pCDNA-vector showed significantly low baseline apoptosis rates, whereas overexpression of RARRES1 noticeably increased the rate of cell apoptosis (Fig. 8A-B). RARRES1 has been reported to activate p53, thereby promoting apoptosis. We subsequently investigated the impact of RARRES1 on p53 in A375 cells, and the results showed that RARRES1 overexpression significantly increased the protein levels of p53 and its target gene BAX. Additionally, we observed an increase in the expression of BAK, another key executor of mitochondrial membrane permeabilization in the mitochondrial pathway apoptosis (Fig. 8C-D). Since ROS plays a crucial role in various signaling pathways, such as cell cycle regulation and apoptosis, we further explored the effect of RARRES1 on ROS and observed a significant increase in ROS levels with RARRES1 overexpression (Fig. 8E-F). As ROS elevation also promotes autophagy^25^, we next accessed whether Rarres1 over-expression impacts autophagy. As shown in Fig. 8G-H, an augmented LC3 conversion from LC3-I to LC3-II in cells overexpressing RARRES1 was discovered through immunoblotting assays. These findings indicate that the overexpression of RARRES1 led to the accumulation of autophagosomes in A375 cells. This could be attributed to either an enhancement in autophagy induction or a suppression in autophagosome degradation. To elucidate the underlying factor contributing to the autophagosome accumulation, we initially assessed the expression levels of key autophagy-related proteins. Our results demonstrated that RARRES1 overexpression significantly upregulated the mRNA expression of ATG5, ATG7, and ATG10 (Fig. 8I), demonstrating that RARRES1 overexpression induced autophagic activation. Next, we investigated whether RARRES1 overexpression hinders downstream processes in autophagic flux. We analyzed the expression of the SQSTM1/P62 protein, which acts as a selective autophagy receptor and is degraded alongside ubiquitinated substrates during autolysosome degradation. As demonstrated in Fig. 8J-K, we observed an accumulation of p62 upon Rarres1 overexpression. This finding aligned with the inhibition of autophagic flux during the later stages. Subsequently, we hypothesized that the blockage of autophagic flux could be attributed to lysosomal dysfunction. Since a low lysosomal pH is essential for maintaining proper lysosomal function, we utilized Lysosensor DND-189, a pH-dependent probe that exhibits increased fluorescence upon acidification, to assess lysosomal pH levels. As presented in Fig. 8L-M, Rarres1 overexpression led to a reduction in fluorescence, indicating a decrease in lysosomal acidification and thus contributing to the impairment of autophagic flux. Overall, these findings suggest that Rarres1 overexpression stimulated autophagic initiation while simultaneously impeding the degradation of autophagic cargo during the later stages.

In summary, our findings suggest that RARRES1 overexpression inhibits SKCM cell proliferation by inducing ROS-mediated cell cycle arrest, apoptosis induction, and autophagy blockade. Furthermore, RARRES1 overexpression also suppresses the migration of SKCM cells (Fig. 8N).

### Overexpression of RARRES1 suppresses tumor growth in vivo

**Figure 9:**
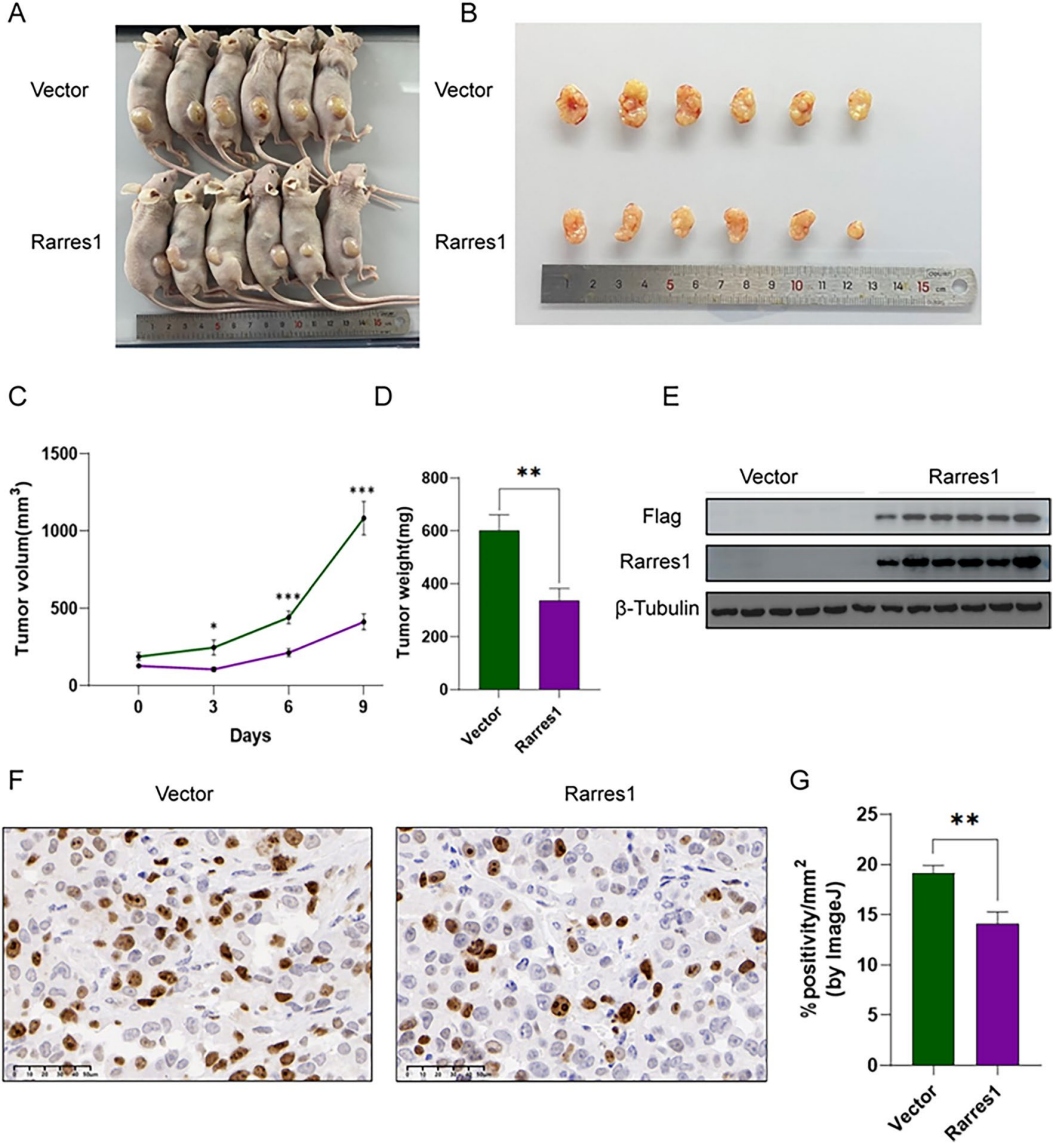
Overexpression of RARRES1 suppresses tumor growth in vivo. (**A**) Pictures of nude mice implanted with A375 cells overexpressing RARRES1. (**B**) Pictures of the tumors harvested at the end of the experiment. (**C**) Tumor growth of nude mice (n = 6). (**D**) The weight of tumors harvested (n = 6). (**E**) Validation of RARRES1 overexpression in tumor tissues by Western blotting. (**F**) Immunohistochemistry staining of Ki-67 in xenograft tumor samples (n = 6). Bar length = 50 μm. (**G**) Quantification of Ki-67 staining. *P < 0.05, **P < 0.01, ***P < 0.001.

Xenograft tumor experiments were conducted to assess the in vivo effects of RARRES1. The nude mice were administered a subcutaneous injection of stable RARRES1-overexpressing cells. The tumors of RARRES1-overexpressing cells exhibited smaller volume and reduced weight (Fig. 9A-D). The stable overexpression of RARRES1 is depicted in Fig. 9E. Immunohistochemical staining (IHC) demonstrated a significant reduction in the expression of the cell proliferation marker Ki67 in the RARRES1 overexpression group (Fig. 9F-G). These results confirmed that RARRES1 overexpression inhibits tumor growth in vivo, consistent with the in vitro experimental findings.

## Discussion

RARRES1, also referred to as Tazarotene-induced gene 1 (TIG1), plays a crucial role in the regulation of various tumorigenic processes. Studies have shown a significant downregulation of RARRES1 expression in several types of cancers, including prostate, colorectal, nasopharyngeal, gastric, endometrial, and testicular cancers. Furthermore, it has been observed that the expression level of RARRES1 is inversely correlated with patient survival in cases of prostate and colorectal cancers^9,10,26–29^. Nonetheless, RARRES1 might have a promotive effect on cancer development in specific tumor types. For example, Wang X et al. demonstrated that enhanced RARRES1 expression in inflammatory breast cancer facilitated the growth of tumors^30^.

To elucidate the expression and functional roles of RARRES1 in SKCM, we obtained data from both the GEO and TCGA databases, followed by comprehensive bioinformatic analyses. Our findings revealed a significant downregulation of RARRES1 expression in SKCM. Furthermore, we observed that low RARRES1 expression was associated with unfavorable clinical features and prognostic outcomes, regardless of whether patients received immunotherapy. Consequently, RARRES1 emerged as an independent prognostic factor for SKCM. Moreover, it held promise as a valuable diagnostic marker. These results substantiated the suppressive role of RARRES1 in the progression of SKCM, supporting previous studies exploring RARRES1 in other tumor types^9,10^.

A recent study has unveiled that the coexistence of tumor-associated CD8^+^ T cells and CD20^+^ B cells is correlated with enhanced survival in metastatic melanoma^31^. Srour et al. performed immunohistochemical staining on samples obtained from melanoma patients receiving immune checkpoint inhibitor. The results of their study revealed a significant association between immune infiltration and the therapeutic efficacy of the treatment^32^. In our study, we conducted GO, KEGG, and GSEA enrichment analysis, which revealed a significant association between RARRES1 expression and the tumor immune microenvironment, cell cycle regulation, migration, apoptosis, autophagy, and oxidative damage. Furthermore, our findings indicated a positive correlation between RARRES1 expression in SKCM and the infiltration of various immune cells, including macrophages, CD8^+^ T cells, B cells, and T helper cells. These results suggest that RARRES1 may enhance its anti-tumor effect in SKCM by promoting immune cell infiltration. Consistent with our findings, a previous study demonstrated that RARRES1 induces M1 macrophage activation to exert antitumor effects in renal clear cell carcinoma^11^.

We also investigated the underlying mechanism behind the reduced expression of RARRES1 in SKCM. Prior research has established a correlation between the transcriptional silencing of RARRES1 in esophageal, gastric, endometrial, and prostate cancers and epigenetic inactivation resulting from promoter hypermethylation^23,28,29,33^. In accordance with the aforementioned studies, our findings demonstrated a notably increased level of RARRES1 promoter methylation in metastatic melanoma when compared to the control group. Moreover, we identified 10 promoter sites that exhibit higher levels of methylation in SKCM as compared to nevus. Additionally, we observed that various copy number mutations, particularly shallow deletion, have also been associated with decreased expression of RARRES1 in SKCM.

Based on the findings of bioinformatic analysis, we proceeded with experimental validation in order to corroborate the expression and function of RARRES1 in SKCM. IHC staining showed a significant downregulation of RARRES1 in SKCM compared to intradermal nevus. Both in vitro and in vivo experiments consistently demonstrated that increased expression of RARRES1 suppressed the proliferation of A375 cells, which aligns with the observations made in a prior study conducted on colorectal cancer cells^9^. Through flow cytometry analysis, we discovered that RARRES1 exerts inhibitory effects on cell proliferation by suppressing the G1/S transition, as evidenced by the downregulation of Cyclin D1 and Cyclin E1 expression. According to the results of the enrichment analysis, it was found that RARRES1 is strongly correlated with the migration and invasion of melanoma. Therefore, a thorough examination was conducted to investigate the influence of RARRES1 on cell migration. Consistent with the initial hypothesis, the outcomes of the transwell assay demonstrated that overexpression of RARRES1 effectively impeded the migration of A375 cells, which was accompanied by a notable elevation in the protein levels of E-cadherin.

As previously mentioned, the upregulation of RARRES1 in glomerular disease resulted in the upregulation of genes related to the cell death pathway. Moreover, the overexpression of RARRES1 in podocytes induced the activation of P53, leading to apoptosis and promoting the advancement of glomerular disease. These discoveries implyed that RARRES1 might have a significant involvement in the process of apoptosis^34^. As expected, our results revealed that RARRES1 overexpression induced apoptosis in A375 cells through upregulating the expression of pro-apoptotic factors P53, BAK and BAX. In addition, studies have demonstrated that ROS plays a key role in apoptosis^25,35^. Consistently, we also found that RARRES1 overexpression promoted ROS production, providing another plausible explanation for RARRES1's role in facilitating cell apoptosis.

On the other hand, we found autophagy flux in A375 cell was inhibited by RARRES1 overexpression. Autophagy is an extensively conserved lysosomal degradation pathway that plays a crucial role in maintaining cellular homeostasis by clearing protein aggregates and damaged organelles^36^. Previous studies have shown that RARRES1 activates autophagy to inhibit tumor progression in prostate cancer and cervical cell carcinoma^7,37^. In our study, we discovered that the overexpression of RARRES1 led to an increase in the expression of ATG5/7/10 and LCII, suggesting the activation of autophagy during the early stages. However, we also observed the accumulation of P62, a biomarker associated with the late stages of autophagy. This accumulation indicated that the overexpression of RARRES1 inhibited the progression of autophagy towards the later stages. Recent research has highlighted that the inhibition of autophagy at later stages can lead to the excessive buildup of undegraded materials within autophagic vacuoles, ultimately resulting in an accelerated process of cell death^38^. Therefore, it was hypothesized that the inhibition of autophagic flux is significantly correlated with the heightened prevalence of cellular apoptosis.

## Conclusion

In conclusion, the expression of RARRES1 in SKCM was found to be diminished as a result of copy number mutations and high promoter methylation. Additionally, it was observed that low RARRES1 expression correlated with a poorer overall survival. Enrichment analysis suggested that RARRES1 may function as a tumor-suppressive factor by regulating various aspects of immune cell infiltration, proliferation, migration, apoptosis, and autophagy. Experimental validation further confirmed the functional roles of RARRES1 in proliferation, migration, apoptosis, and autophagy. This study emphasizes the significance of RARRES1 as a valuable prognostic biomarker for SKCM and highlights its potential as a promising therapeutic target.

### Supplementary Information

Supplementary Table 1.

Supplementary Table 2.

## Data Availability

The datasets featured in this study are available in online repositories, and the names of these repositories along with their respective accession numbers can be located within the article. The GSE15605 and GSE120878 datasets were downloaded from the GEO (https://www.ncbi.nlm.nih.gov/geo/) database. Patient information and prognosis data of SKCM was obtained from TCGA (https://www.cancer.gov/ccg/research/genome-sequencing/tcga).

## Abbreviations

SKCM: Skin cutaneous melanoma
RARRES1: Retinoic acid receptor responder 1
TCGA: The Cancer Genome Atlas
GO: Gene ontology
KEGG: Kyoto encyclopedia of genes and genomes
TIG1: Tazarotene-induced gene
IHC: Immunohistochemistry
ROC: Receiver operating curve
GEO: Gene expression omnibus
BP: Biological processes
MF: Molecular functions
CC: Cellular components
FDR: False discovery rate
CNV: Copy number variants
OS: Overall survival
DSS: Disease specific survival
HR: Hazard ratio
AUC: Area under the curve
IDN: Intradermal nevus
